# Improving National and International Surveillance of Movement Behaviours in Childhood and Adolescence: An International Modified Delphi Study

**DOI:** 10.1007/s40279-024-02104-2

**Published:** 2024-10-03

**Authors:** John J. Reilly, Rachel Andrew, Chalchisa Abdeta, Liane B. Azevedo, Nicolas Aguilar Farias, Sharon Barak, Farid Bardid, Bruno Bizzozero-Peroni, Javier Brazo-Sayavera, Jonathan Y. Cagas, Mohamed-Souhaiel Chelly, Lars B. Christiansen, Visnja D. Djordjic, Catherine E. Draper, Asmaa El-Hamdouchi, Elie-Jacques Fares, Aleš Gába, Kylie D. Hesketh, Mohammad Sorowar Hossain, Wendy Huang, Alejandra Jáuregui, Sanjay K. Juvekar, Nicholas Kuzik, Richard Larouche, Eun-Young Lee, Sharon Levi, Yang Liu, Marie Löf, Tom Loney, José Francisco López-Gil, Evelin Mäestu, Taru Manyanga, Clarice Martins, Maria Mendoza-Muñoz, Shawnda A. Morrison, Nyaradzai Munambah, Tawonga W. Mwase-Vuma, Rowena Naidoo, Reginald Ocansey, Anthony D. Okely, Aoko Oluwayomi, Susan Paudel, Bee Koon Poh, Evelyn H. Ribeiro, Diego Augusto Santos Silva, Mohd Razif Shahril, Melody Smith, Amanda E. Staiano, Martyn Standage, Narayan Subedi, Chiaki Tanaka, Hong K. Tang, David Thivel, Mark S. Tremblay, Edin Uzicanin, Dimitris Vlachopoulos, E. Kipling Webster, Dyah Anantalia Widyastari, Pawel Zembura, Salome Aubert

**Affiliations:** 1 Department of Psychological Sciences and Health, University of Strathclyde, Glasgow, Scotland; 2 School of Education, University of Wollongong, Wollongong, Australia; 3 School of Sport and Physical Activity, Sheffield Hallam University, Sheffield, UK; 4 Department of Physical Education, Sports and Recreation, Universidad de La Frontera, Temuco, Chile; 5 Department of Nursing, Faculty of Health Science, Ariel University, Ariel, Israel; 6 Institute of Education, University of Strathclyde, Glasgow, Scotland; 7 Instituto Superior de Educación Física, Universidad de La República, Rivera, Uruguay; 8 Health and Social Research Center, Universidad de Castilla-La Mancha, Cuenca, Spain; 9 Department of Sports and Computer Science, Universidad Pablo de Olavide, Seville, Spain; 10 PDU EFISAL, Centro Universitario Regional Noreste, Universidad de La República, Montevideo, Uruguay; 11 Department of Sports Science, College of Human Kinetics, University of the Philippines Diliman, Quezon, Philippines; 12 Research Laboratory (LR23JS01) «Sport Performance, Health and Society», Higher Institute of Sport and Physical Education of Ksar Saîd, University of Manouba, Tunis, Tunisia; 13 Department of Sport Sciences and Clinical Biomechanics, University of Southern Denmark, Odense, Denmark; 14 Faculty of Sport and Physical Education, University of Novi Sad, Novi Sad, Serbia; 15 Faculty of Health Sciences, SAMRC Developmental Pathways for Health Research Unit, University of the Witwatersrand, Johannesburg, South Africa; 16 National Centre for Energy Sciences and Nuclear Techniques, Kenitra, Morocco; 17 American University of Beirut, Beirut, Lebanon; 18 Faculty of Physical Culture, Palacký University Olomouc, Olomouc, Czech Republic; 19 Institute of Physical Activity and Nutrition, Deakin University, Burwood, Australia; 20 Biomedical Research Foundation, Dhaka, Bangladesh; 21 Independent University, Dhaka, Bangladesh; 22 Department of Sport, Physical Education and Health, Hong Kong Baptist University, Hong Kong, China; 23 Department of Physical Activity and Healthy Lifestyles, National Institute of Public Health, Cuernavaca, México; 24 Vadu Rural Health Program, KEM Hospital Research Centre, Pune, India; 25 Children’s Hospital of Eastern Ontario Research Institute, Ottawa, ON K1H 5B2 Canada; 26 Faculty of Health Sciences, University of Lethbridge, Lethbridge, Canada; 27 School of Kinesiology and Health Studies, Queen’s University, Kingston, Canada; 28 Efsharibari- National Program for Active and Healthy Living, Ministry of Health, Haifa, Israel; 29 School of Public Health, University of Haifa, Haifa, Israel; 30 School of Physical Education, Shanghai University of Sport, Shanghai, China; 31 Department of Biosciences and Nutrition, Karolinska Institutet, Solna, Sweden; 32 College of Medicine, Mohammed Bin Rashid University of Medicine and Health Sciences, Dubai Health, Dubai, United Arab Emirates; 33 One Health Research Group, Universidad de Las Américas, Quito, Ecuador; 34 Institute of Sport Sciences and Physiotherapy, Faculty of Medicine, University of Tartu, Tartu, Estonia; 35 Division of Medical Sciences, University of Northern British Columbia, Prince George, Canada; 36 Faculty of Sports, Research Centre of Physical Activity, Health and Leisure, Porto, Portugal; 37 Laboratory for Integrative and Translational Research in Population Health (ITR), University of Porto, Porto, Portugal; 38 Faculty of Sport Sciences, University of Extremadura, Badajoz, Spain; 39 Human Potential Translational Research Programme, Yong Loo Lin School of Medicine, National University of Singapore, Singapore, Singapore; 40 Faculty of Medicine and Health Sciences, University of Zimbabwe, Harare, Zimbabwe; 41 Centre for Social Research, University of Malawi, Zomba, Malawi; 42 Discipline of Biokinetics, Exercise and Leisure Sciences, College of Health Sciences, University of KwaZulu-Natal, Durban, South Africa; 43 School of Education and Leadership Studies, University of Ghana, Legon, Ghana; 44 School of Health and Society, University of Wollongong, Wollongong, Australia; 45 Human Kinetics and Health Education, University of Lagos, Lagos, Nigeria; 46 Institute for Physical Activity and Nutrition, School of Exercise and Nutrition Sciences, Deakin University, Burwood, Australia; 47 Centre for Community Health Studies (ReaCH), Faculty of Health Sciences, University Kebangsaan Malaysia, Kuala Lumpur, Malaysia; 48 Evelyn H. Ribeiro, Physical Activity Epidemiology Group at the University of Sao Paulo (GEPAF/USP), Sao Paulo, Brazil; 49 Department of Physical Education, Sports Center, Federal University of Santa Catarina, Santa Catarina, Brazil; 50 Centre for Healthy Ageing and Wellness (HCARE), Universiti Kebangsaan Malaysia, Bandar Baru Bangi, Malaysia; 51 School of Nursing, The University of Auckland, Auckland, New Zealand; 52 Pennington Biomedical Research Center, Louisiana State University, Louisiana, USA; 53 Centre for Motivation and Health Behaviour Change, Department for Health, University of Bath, Bath, UK; 54 The Lown Scholar, Harvard T.H. Chan School of Public Health, Harvard University, Cambridge, USA; 55 Department of Human Nutrition, Tokyo Kasei Gakuin University, Tokyo, Japan; 56 Faculty of Public Health, Pham Ngoc Thach University of Medicine, Ho Chi Minh City, Vietnam; 57 Laboratory of the Metabolic Adaptations to Exercise under Physiological and Pathological Condition (AME2P UPR3533), International Research Chair “Health in Motion”, Clermont Auvergne University, Clermont-Ferrand, France; 58 Healthy Active Living and Obesity Research Group, Children’s Hospital of Eastern Ontario Research Institute, Ottawa, ON Canada; 59 Faculty of Physical Education and Sport, University of Tuzla, Tuzla, Bosnia-Herzegovina; 60 Department of Sport and Health Science, University of Exeter, Devon, UK; 61 Department of Kinesiology, Recreation, and Sport Studies, University of Tennessee, Knoxville, USA; 62 Institute for Population and Social Research, Mahidol University, Nakhon Pathom, Thailand; 63 Jozef Pilsudski University of Physical Education, Warsaw, Poland; 64 Active Healthy Kids Global Alliance, Healthy Active Living and Obesity Research, Ottawa, Canada; 65 Department of Occulational and Physical Therapy, University of Namibia, Windhoek, Namibia

## Abstract

**Background:**

The actions required to achieve higher-quality and harmonised global surveillance of child and adolescent movement behaviours (physical activity, sedentary behaviour including screen time, sleep) are unclear.

**Objective:**

To identify how to improve surveillance of movement behaviours, from the perspective of experts.

**Methods:**

This Delphi Study involved 62 experts from the SUNRISE International Study of Movement Behaviours in the Early Years and Active Healthy Kids Global Alliance (AHKGA). Two survey rounds were used, with items categorised under: (1) funding, (2) capacity building, (3) methods, and (4) other issues (e.g., policymaker awareness of relevant WHO Guidelines and Strategies). Expert participants ranked 40 items on a five-point Likert scale from ‘extremely’ to ‘not at all’ important. Consensus was defined as > 70% rating of ‘extremely’ or ‘very’ important.

**Results:**

We received 62 responses to round 1 of the survey and 59 to round 2. There was consensus for most items. The two highest rated round 2 items in each category were the following; for funding (1) it was greater funding for surveillance and public funding of surveillance; for capacity building (2) it was increased human capacity for surveillance (e.g. knowledge, skills) and regional or global partnerships to support national surveillance; for methods (3) it was standard protocols for surveillance measures and improved measurement method for screen time; and for other issues (4) it was greater awareness of physical activity guidelines and strategies from WHO and greater awareness of the importance of surveillance for NCD prevention. We generally found no significant differences in priorities between low-middle-income (*n* = 29) and high-income countries (*n* = 30) or between SUNRISE (*n* = 20), AHKGA (*n* = 26) or both (*n* = 13) initiatives. There was a lack of agreement on using private funding for surveillance or surveillance research.

**Conclusions:**

This study provides a prioritised and international consensus list of actions required to improve surveillance of movement behaviours in children and adolescents globally.

**Supplementary Information:**

The online version contains supplementary material available at 10.1007/s40279-024-02104-2.

## Key Points

**Table:** 

This study used a Delphi process with 62 international experts in child and adolescent movement behaviours to identify the actions needed to improve global surveillance of movement behaviours in childhood and adolescence.
There was a high degree of agreement for almost all items in the Delphi Survey. The top priority was increased funding for surveillance – this would underpin the other priority actions identified: establishment of regional hubs to support surveillance, development of standardised surveillance protocols, improved measurement methods, improved human capacity in surveillance of human movement behaviours, and greater stakeholder awareness of World Health Organisation (WHO) movement behaviour guidelines and strategies.
Respondents did not reach consensus on where the funding of surveillance should come from. There may be a need to establish a globally accepted framework for using funding from private and public sources for movement behaviour research and surveillance.

## Introduction

The monitoring of health behaviours and/or health outcomes, usually in national surveys – ‘surveillance’ – is a fundamental pillar of public health [1–4]. The surveillance of movement behaviours (physical activity, PA; sedentary behaviour and sleep) is essential for many reasons: to provide an understanding of the extent to which guidelines are being met; to allow identification of inequalities and temporal trends, to evaluate the effects of policy or other environmental changes (such as the impact of movement restrictions to limit the spread of infectious disease), to allocate resources appropriately and to permit cross-country comparisons [1, 3]. The importance of movement behaviours to the current and future health of children and adolescents makes surveillance vital. Evidence-based guidelines now exist for healthy levels of screen time, sleep duration and time spent in physical activity for children under 5 and for school-age children and adolescents [5–10]. A renewed focus on improving surveillance of the movement behaviours in children and adolescents is appropriate because PA levels were typically well below the World Health Organization (WHO) guidelines prior to the coronavirus disease 2019 (COVID-19) pandemic [11–13] and declined during the pandemic [14–16], while time spent in sedentary behaviour increased during the pandemic [17–19].

Despite the importance of movement behaviour surveillance in childhood and adolescence globally, surveillance has focused on physical activity only (with no surveillance or very limited surveillance of sedentary behaviour and sleep in many countries), and surveillance of physical activity has been characterised as inadequate [1, 3]. The review of international physical activity surveillance by Aubert et al. [3] found eight inter-continental surveillance systems for physical activity in children and/or adolescents. None of these had data on those under 5 years, few had data on those under 10 years, only two had a track record of continuity/sustainability, and all tended to under-represent minorities (such as rural dwellers, those not attending school, those with chronic disease or disability) and under-represent or exclude those from low-and-middle-income countries (LMICs)[3]. Aubert et al. [3] also found that, even where surveillance data were available, the information provided was often very limited, derived from methods which may be invalid/unreliable and culturally inappropriate in many settings [1, 3], and based on small and often unrepresentative samples, with the most recent data collection over a decade ago. Methodological difficulties and uncertainties have contributed to limitations in surveillance. For example, there are marked differences in time spent in physical activity between surveillance initiatives derived from self- or parent-report versus those from device-based measures [1, 3], and regardless of the method used, cross-country comparisons and national physical activity level rankings vary substantially across intercontinental surveillance systems findings [3].

In a recent paper based on the experience from the Active Healthy Kids Global Alliance (AHKGA) initiative [1], the components of high-quality movement behaviour surveillance were described. This encompassed surveys carried out regularly on a sustainable basis and frequent enough to be informative; inclusive of all ages from birth to the end of adolescence; nationally representative; large enough to identify inequalities (by age, gender, socio-economic status, ethnic group, urban or rural setting, chronic disease and disability) and with the inclusion of LMICs as well as high-income countries (HICs); use of valid, reliable and culturally appropriate methods of measurement of physical activity, sedentary behaviour and sleep; and results interpreted and reported accurately and made available to all stakeholders reasonably soon after data collection. While that paper attempted to describe what high-quality surveillance would consist of [1] there is a currently a dearth of evidence on how to achieve it, i.e. how to transform existing surveillance efforts into more robust surveillance systems. In other words, previous studies identified a number of problems with surveillance systems globally, but did not identify the solutions to these problems, or the extent to which the surveillance research community globally would agree on and prioritise solutions.The aim of the present study was therefore to identify what actions are required to improve current movement behaviour surveillance to provide much more comprehensive and accurate national and international surveillance in future.

## Methods

### Participants and Recruitment

Participants in the present study were involved in one or both of the major long-term international movement behaviour surveillance initiatives for young children and school-age children adolescents, i.e. the International Study of Movement Behaviours in the Early Years (SUNRISE) [20] and the AHKGA [11, 21], respectively. The SUNRISE study was formed in 2018 with the primary scientific aims of estimating the global prevalence of meeting physical activity, sedentary behaviour and sleep guidelines for 3- and 4-year-olds and identifying the correlates of meeting those guidelines. Other aims included building global capacity in surveillance of movement behaviours in the early years [20]. To estimate the prevalence of meeting guidelines in SUNRISE, a standardised protocol has been established which combines device-based measurement and parent-report, piloted in urban and rural settings in participating countries [20]. To avoid over-representation of high-income countries (HICs), entry to SUNRISE was initially restricted so that LMICs would form at least 60% of participating countries [20]. The SUNRISE initiative is co-ordinated from the University of Wollongong in Australia, but led by a leadership group which is balanced concerning gender, age and country level of economic development [20], and it currently includes over 70 participating countries. The SUNRISE initiative has no core/permanent funding and is not currently a sustainable intercontinental surveillance system. The University of Wollongong has Australian grant funding for the study until the end of 2025, and participating countries have had variable success in funding pilot studies with samples of approximately 100 children and main studies samples of around 1000 children.

The AHKGA Global Matrix initiative has involved around 70 countries and jurisdictions since its inception in 2014. The primary scientific aims of the AHKGA are to report on multiple indicators of physical activity and sedentary behaviour, and the influences on those behaviours internationally [11], with a view to prompting improvements in physical activity and sedentary behaviour. The AHKGA Global Matrix also aims to build capacity in child and adolescent physical activity and sedentary behaviour surveillance and promotion globally [21]; it is led by an international board balanced with respect to gender, age and country level of economic development. The AHKGA Global Matrix reports data on school-age children and adolescents (5–17-year-olds), typically every 2–4 years, but many of the participating countries also report data on those under 5 years. Data are summarised as report card grades for each indicator, based on a harmonised development protocol. Each report card team grades the best available data for each indicator, using a standardized grading scheme and harmonized indicator benchmarks then submit its grades for external peer review from AHKGA. Grades can be derived using either or both self/parent report or device-based measurement, so long as the method is deemed appropriate for the population in which it is being used and with no major bias. The AHKGA Global Matrix has no core funding as an international surveillance initiative and depends on a combination of research and knowledge exchange grants and support in kind at national and international level.

**Fig. 1:**
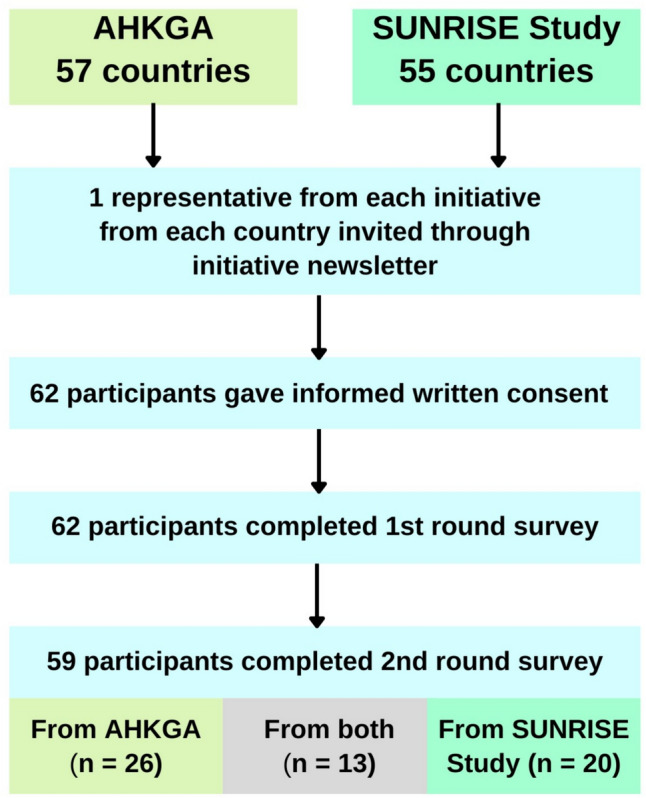
Flow chart describing recruitment of participants. AHKGA, Active Healthy Kids Global Alliance; SUNRISE, International Surveillance Study of Movement Behaviours in the Early Years

Both the SUNRISE and AHKGA initiatives included 50–70 actively participating countries when the present study started recruitment at the end of 2022. In some countries and jurisdictions, the leads/co-leads for SUNRISE and the AHKGA were the same individuals. To avoid excessive representation in the sample from particular countries, we capped participation to one representative per country per initiative so that the maximum response from any participating country came from two individuals, one representing SUNRISE and one representing the AHKGA (see Fig. 1). We invited and permitted participation only from leads and/or co-leads of each initiative to ensure that participants had enough expertise in movement behaviour surveillance.

To recruit participants, a brief news item was included in the December 2022 e-newsletters of SUNRISE and the AHKGA. This news item explained the purpose of the study and the expectations of study participants, it included links to relevant publications [1, 3], links to the study information and consent forms, and contact details for the lead researchers and a link to round 1 of the survey. The surveys asked participants to provide information about their sociodemographic (e.g. nationality and gender) and professional characteristics (e.g. current field of work and type of organisation). All participants gave electronic informed written consent and were aware that participation was voluntary and that they could withdraw at any point during the survey without any consequences. The study was approved by the University of Strathclyde, Glasgow Scotland, School of Psychological Sciences and Health Research Ethics Committee (reference 03.05.10.2022).

### Leadership Group, Development of the Survey and Pilot Study

Developing the survey involved pre-identifying items for expert consideration and categorising those items. A leadership group of 12 authors from one or both of the SUNRISE Study or the AHKGA, representing countries across a range of geographical areas and economic development (C.A., Ethiopia; R.A., Scotland; S.A., France and Pacific; J.Y.C., Philippines; R.L., Canada; S.K.L., Israel; N.M., Zimbabwe; A.D.O., Australia; J.J.R., Scotland; C.T., Japan; M.T., Canada; D.A.W., Thailand), developed the survey items and item categories. This process involved J.J.R. and S.A. using the published critique of current international surveillance [3] to identify potential solutions to all major problems identified in that critique [3], and then operationalising the potential solutions to each problem as an initial list of potential survey questions and categories. To finalise the survey questions and categories, this initial list was shared with all members of the leadership group in an email – the leadership group discussed online/provided comments via email until there was agreement on the content of the survey, i.e. the questions and categories which would be included. To ensure optimal survey methods, four members of the leadership group piloted two different survey options (Word Forms versus Qualtrics). Additionally, they piloted two different response scales (three-point versus five-point Likert responses). Following the pilot, the four members of the leadership group shared their views with the rest of the leadership group and there was unanimous agreement that Qualtrics should be used for the present study as it was slightly easier to use than Word Forms and provided better access to numerical data and plots. The five-point Likert scale was chosen because it permitted a more nuanced set of options for respondents than the three-point scale: a wider range of response options was considered more informative, i.e. more likely to identify subtle differences in respondent preferences.

### Delphi Study

This study used an online modified Delphi process [22–25]. The modification to the original Delphi technique was that the items which participants were asked to rate were identified in advance and classified into the four categories by the Leadership Group (Funding; Capacity Building; Methods; Other Issues) as described above. Each category in the survey contained open questions to allow respondents to suggest additional survey items, recommend removal of items, and to provide more detailed responses. The main goals of this Delphi study were to identify whether and where consensus among experts was achieved, and to identify respondent priorities [22–25]. Agreement of > 70% of participants is widely regarded as indicative of consensus [23] and was used in the present study. The number of survey rounds required in a Delphi Study varies, but often depends on when consensus is reached [22–25]. In the present study, consensus was reached for most survey items following round 1, but a second survey (round 2) was offered so that participants could reflect on their responses with knowledge of the responses from their peers. Data collection was stopped after round 2 of the survey due to consensus being reached for most survey items, including those rated as highest priority, and the present study conclusions are based on round 2 results. Although high participant retention between survey rounds is considered important to the rigour of Delphi Studies, the precise degree of retention required is unclear. It has been argued that retention of at least 70% between survey rounds ensures rigour of the Delphi technique [24], so efforts were made to achieve at least a 70% retention rate by using reminders to participants (one email reminder for Round 1, and two reminders for Round 2).

The survey was provided in English only (Supplementary File) and consisted of 40 close-ended questions (some of which had several parts, giving 52 questions in round 1 and 53 in round 2), split into four categories: (1) funding; (2) capacity building; (3) methods; (4) other issues (e.g. greater policymaker awareness of movement behaviour guidelines, links to non-communicable disease (NCD) prevention). Participants were asked to rank each item on the five-point Likert scale: ‘extremely important’ (5, highest), ‘very important’, ‘moderately important’, ‘slightly important’, ‘not at all important’ (1, lowest). ‘Don’t know/prefer not to say’ was also offered as an option. Round 1 of the survey was open for 4 weeks from mid-December 2022 until mid-January 2023, with a reminder email sent to participants 1 week before the deadline. In round 2, participants were emailed a summary of the responses to round 1 that included the top ten endorsed items and bottom five rated items, the ratings for all 52 items, and a summary of responses to the open questions***.*** Participants were asked to read this documentation before completing round 2. Round 2 was opened for 5 weeks from mid-February 2023 until the end of March 2023, and reminder emails were sent 1 and 2 weeks before the deadline.

### Data Analysis and Interpretation

Descriptive statistics were used to report the participants’ characteristics, including gender, occupation, level of economic development of the country they represented and organisation (SUNRISE, AHKGA or both). Summary responses are provided in the Supplementary Files. Responses for all items were converted to numbers (1.0–5.0) for the quantitative analysis. Consensus (agreement) was defined as the achievement of > 70% of the sample rating either ‘extremely important’ or ‘very important’ as noted above.

We examined two potential differences in priority ratings within our sample. First, by country level of economic development – between LMICs versus HICs. We used World Bank 2022–2023 Classifications [26] to classify each participating country by level of economic development and Mann–Whitney *U* tests to assess the statistical significance of median differences in responses between LMICs and HICs. Second, we considered potential median differences in responses between the initiative which participants represented (SUNRISE or AHKGA or both). The significance of median differences in responses between initiatives was tested using Kruskal–Wallis Tests. Jamovi (www.jamovi.org) was used for statistical analyses and a* p* level of 0.05 used to define statistical significance.

Given the substantial amount of data generated and the priority placed on final survey rounds in Delphi studies [22–25], the Results section below focusses on round 2 survey data and analyses. However, data from both rounds of the survey can be found below or in the Supplementary Files.

## Results

### Participant Characteristics

**Fig. 2:**
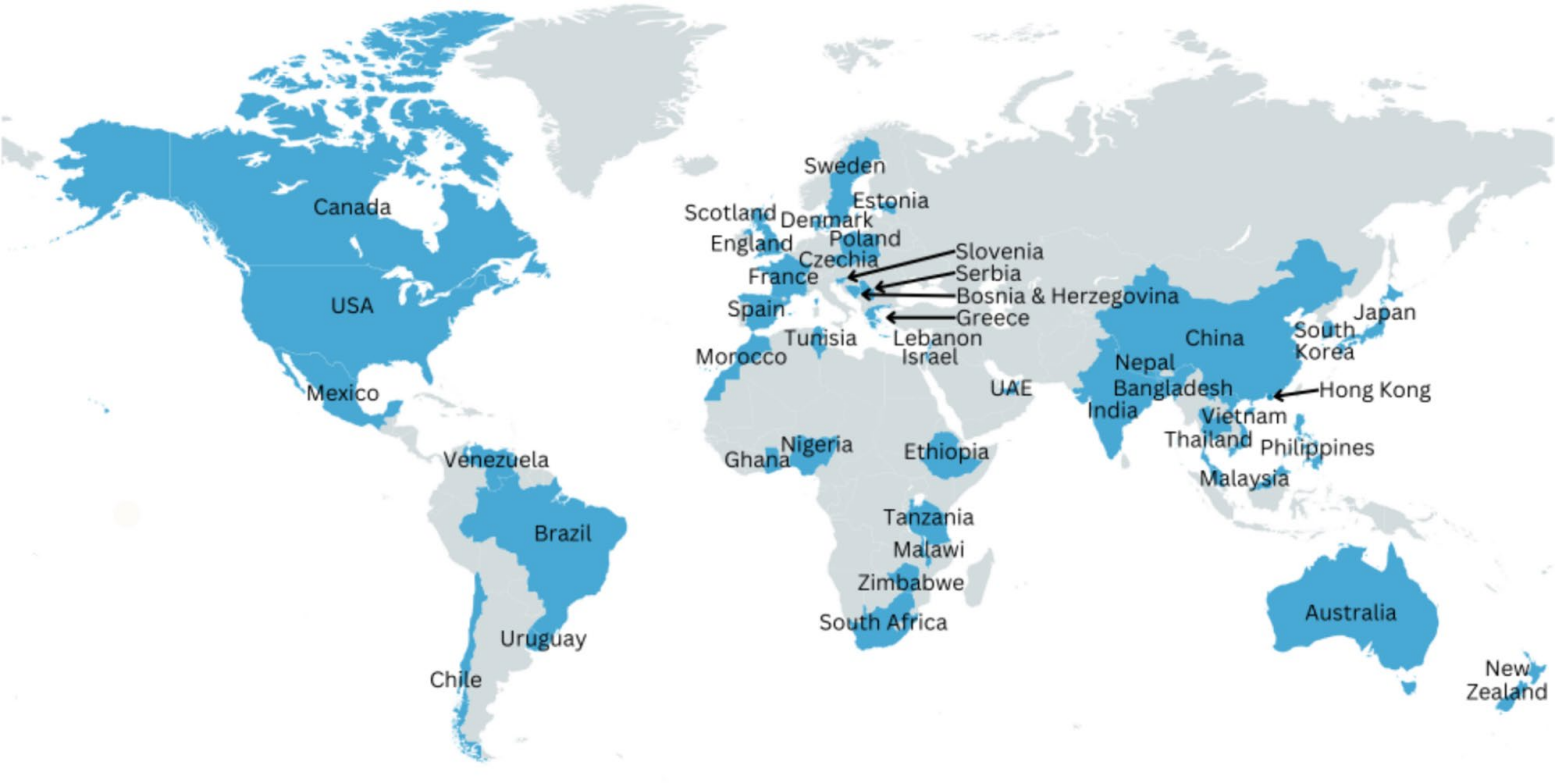
Countries with participant(s) in the present Delphi study

The process of recruitment and retention of participants through the study is summarised in Fig. 1. Participant characteristics are summarised in Table 1 for both survey rounds. A total of 62 participant responses were recorded in round 1 and 59 participants in round 2 (retention rate of 95%). Of the 59 round 2 respondents, 29 were from LMICs and 30 from HICs. The broad extent of global coverage of responses is shown in Fig. 2. Respondents in round 2 were spread across SUNRISE (*n* = 20), AHKGA (*n* = 26) and both (*n* = 13), representing a recruitment rate of approximately 60% of all countries in SUNRISE and AHKGA in 2022. All those who consented to participate completed the survey. The gender split between the respondents was equal, with 29 males and 29 females (1 respondent did not declare gender) in round 2. We did not collect data on career stage/age of study participants, but most respondents worked in research/academia; mostly in established academic posts (*n* = 35) so probably largely mid-career or later, with a smaller number in graduate or doctoral research posts (*n* = 18), or in government and non-governmental organisations, or other posts (*n* = 6).

**Table 1:** Participant characteristics, survey rounds 1 and 2

	Round 1 (*n* = 62)	Round 2 (*n* = 59)
Country status		
Low-middle income	32 (52%)	29 (49%)
High income	30 (48%)	30 (51%)
Gender		
Male	30 (48%)	29 (50%)
Female	32 (52%)	29 (50%)
Initiative		
SUNRISE study	23 (37%)	20 (34%)
AHKGA	26 (42%)	25 (42%)
Both initiatives	13 (21%)	13 (24%)
Working sector		
Academia – graduate or doctoral or postdoctoral researcher	18 (28%)	18(31%)
Academia – established academic post	37 (60%)	35 (59%)
Government/policy	3 (5%)	2 (3%)
Non-governmental organization	3 (5%)	3 (5%)
Other	1 (2%)	1 (2%)

### Participant Responses

**Fig. 3:**
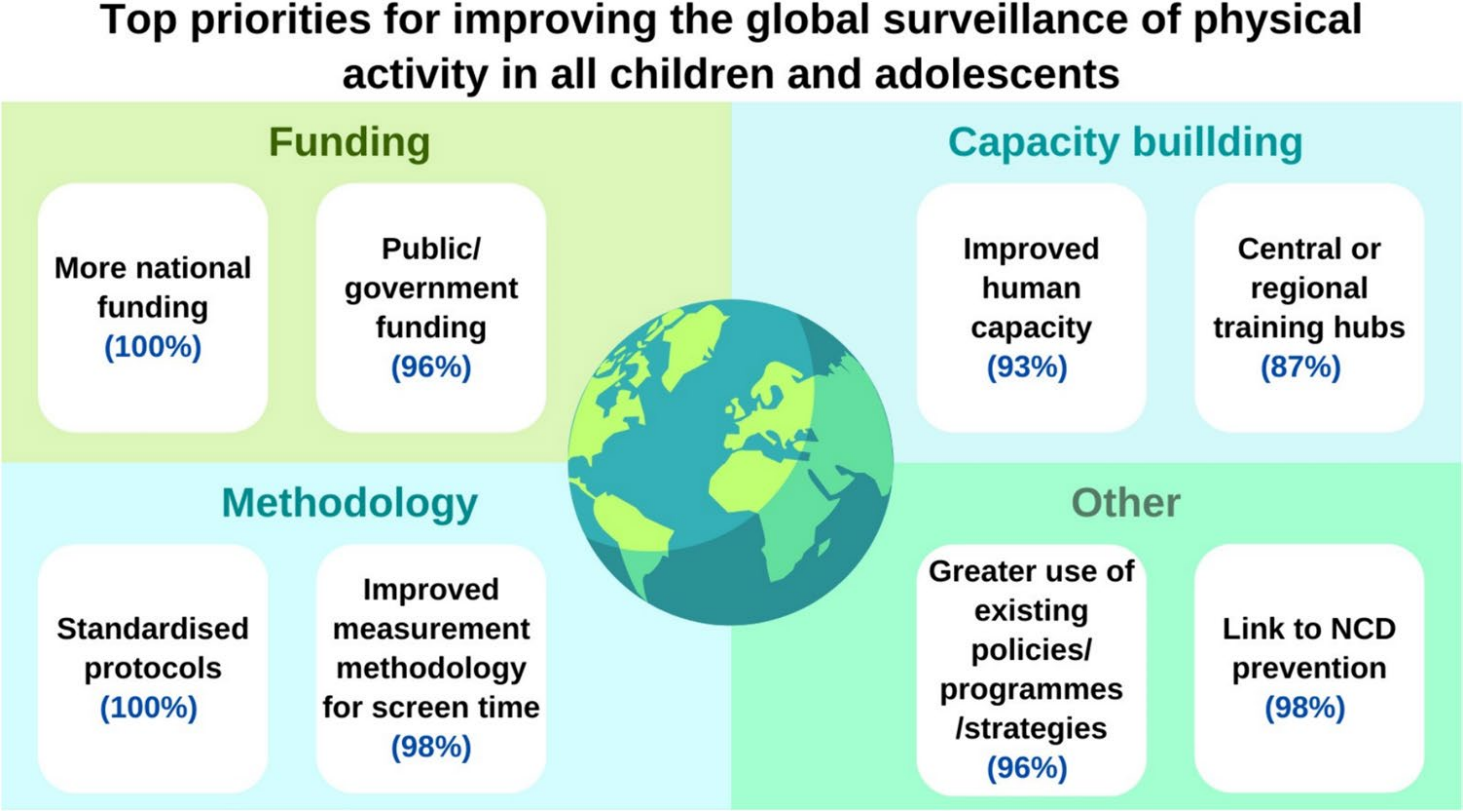
Top two priorities in round 2 for each of the four categories. Percentage agreement for each of the items is reported in blue. NCD, non-communicable diseases

Figure 3 shows the top two priorities in each of the four response categories in round 2 by the highest percentage of agreement. These top priorities were similar in round 1 (Supplementary File). The top priorities from round 2 by category averaged 96% agreement (extremely or very important) and were as follows: funding – more funding for surveillance at national level and public funding sources; capacity building – greater human capacity (knowledge, skills) in physical activity and health as a subject and setting up international or regional hubs to support surveillance; methods – availability of standard protocols for surveillance of movement behaviours and improved methodology for measurement of screen time; and other issues – greater use of ‘soft resources’ (WHO guidelines, policies and strategies for movement behaviours at national level) and advocacy for greater recognition at the national level of the link between child and adolescent movement behaviours and non-communicable disease (NCD) prevention.

Table 2 summarises the top ten rated items, with no restriction on the category of item, in round 2. Agreement for the top ten items overall averaged 97%. The two highest rated priorities in the funding category were included in the top ten priorities overall (increased funding, increased public funding). Four of the top ten items were in the methods category, including the top two rated priorities in this category mentioned above (availability of standard measurement protocols, improved measurement of screen time, surveys large enough to identify inequalities in time spent in the movement behaviours, surveys which are much more inclusive for children and adolescents with disability). Four of the top ten priorities were in the ‘other issues’ category including the top two rated items in this category mentioned above: linkage at national/policy level between the movement behaviours and NCD prevention, and greater use of movement behaviour guidelines and strategies at national levels. The other priority items were greater awareness of the linkage between the movement behaviours and child and adolescent development, and better partnerships at national level between those responsible for surveillance and those with expertise in movement behaviour measurement. The need to improve human capacity in movement behaviours (education, training, research experience) reached 93% agreement. The 70% threshold to define consensus was reached for most of the items in the survey.

**Table 2:** Top ten rated items from all sections of survey round 2, ranked 1 from 1–10

Item	Percentage rating ‘very’ or ‘extremely’ important
***Funding*** More national funding	100
***Methodology*** Standard protocols like SUNRISE	100
***Methodology*** Ability to measure time spent with screens other than television	98
***Other issues*** Linkage of surveillance to NCD prevention	98
***Funding*** Government funding	96
***Other issues*** Greater national use of existing policies/programmes/strategies	96
***Methods*** Surveys large enough to identify inequalities	96
**M*ethods*** Greater inclusion of groups currently under-represented	95
***Other issues*** Better partnerships between surveillance experts in academia and government departments doing surveillance at national level	95
***Other issues*** Linkage of surveillance to child/adolescent development	95

SUNRISE is the International Study of Movement Behaviours in the Early Years. NCD, non-communicable disease

We found no statistically significant differences between ratings from respondents representing LMICs versus HICs for any of the top-rated items (Supplementary File). We found only one statistically significant difference in median responses between respondents representing SUNRISE versus AHKGA versus both initiatives (Supplementary File), which was that the methods item ‘Need to improve measurement of screens other than TV’ was rated very slightly lower in respondents representing SUNRISE than those representing the AHKGA.

The survey item with by far the lowest (26%) agreement that it was ‘very’ or ‘extremely’ important (and the only item with < 30% of respondents rating as ‘very or extremely important’) was obtaining funding for surveillance from private/commercial sources. The open text option for this item also received several conflicting comments. Some respondents were much more concerned than others about using private/commercial funding for surveillance, and/ or for surveillance research, and some also expressed concerns that government funding of surveillance might encourage bias.

## Discussion

### Main Findings and Implications of Study Findings

The present study found an international consensus over the actions required to achieve high- quality national and international surveillance of the movement behaviours (physical activity, sedentary behaviour, sleep) in children and adolescents across diverse countries around the world. The consensus was global, extended across levels of economic development, and across surveillance expertise in early childhood, later childhood and adolescence.

Increased funding at the national level for surveillance was rated as a top priority, highlighting the significant underfunding of movement behaviour surveillance in countries across all levels of economic development, not just in LMICs. While methodological improvements for surveillance were rated as high priorities these are also dependent on increased funding to a large extent. The inadequate size and representativeness of current surveillance systems could be addressed by improved funding. Increased funding will also be required to deal with the highest priority methodological problems, notably the need to develop and validate new ways of measuring screen time: 98% of the sample rated this as either extremely or very important. Respondents prioritised the need for larger and more inclusive surveys so that a proper understanding of inequalities (e.g. among children and adolescents with disability, urban versus rural populations) can be achieved. Surveillance that includes important sub-groups, such as those living with disability, indigenous populations and individuals from LMICs, can help identify and address health inequalities. There was also very high level of agreement on the need to improve measurement of a number of other variables, e.g. physically active play, compliance with muscle-strengthening guidance (Supplementary File). The need for standardised surveillance protocols that can be used across countries was also a high priority.

How the findings of the present study should be implemented is a matter for stakeholders to decide, but a number of priority actionable steps have been identified: research effort/funding should be directed to improved methodology for measurement of screen time, physical activity play and other variables (Supplementary File); advocacy should be used to increase funding for surveillance; regional or global networks for surveillance need to be formed or existing networks need long-term funding so they can be expanded/sustained; a framework for ethical use of government and non-government funding in surveillance should be developed; global capacity in movement behaviour research and surveillance should be increased by greater availability of education and training opportunities and greater opportunities to work with existing surveillance collaborations; new standardised protocols for surveillance should be agreed upon; and advocacy and partnership building at a national level should be undertaken so that the importance of the movement behaviours (for NCD prevention, for example) is understood more widely, and so that awareness of national and international movement behaviour guidelines and strategies is increased.

#### Implications for Future Surveillance

The current model of movement behaviour surveillance within national health surveys in many countries requires re-evaluation and upgrading [1, 3]. The common practice of assessing the time spent in movement behaviours and intensity of physical activity by using non-validated, or even demonstrably invalid [1], questionnaire items within national health surveys has many limitations. There is empirical evidence that this approach has failed in some countries, e.g. by producing misleading data on physical activity levels, with unintended adverse consequences for public health policy [1]. Alternative national and international surveillance approaches are likely to be needed. More device-based surveillance will be useful [24, 25] but device-based measurement is not a simple solution. For example, choices made when processing accelerometry data can have profound consequences for apparent levels of physical activity [3]. For some countries, informative device-based surveillance of physical activity with accelerometers is already well-established [3], and accelerometry can also measure time spent sedentary. Accelerometry cannot measure screen time but can measure time spent sleeping and accelerometer-measured sedentary time may be a proxy for screen time in the absence of more direct and accurate measures. A discussion of the advantages and disadvantages of device-based surveillance of the movement behaviours is beyond the scope of the present manuscript. In summary, devices may be essential for accurate measurement of overall time use and intensity of physical activity. Devices are useful to validate self-reports or parent-proxy reports of the movement behaviours [1, 3] but inherently lack the ability to capture qualitative information regarding the context, type, or purpose of the activity undertaken, restraining the depth of insights available for informing the development of physical activity policies. Devices alone are unlikely to provide comprehensive methods for all surveillance requirements. Devices might also complement subjective methods in surveys.

A risk of the growth of device-based surveillance is that the increased cost relative to subjective methods could lead to increased inequality in global surveillance, with reduced access to devices in LMICs relative to HICs. International movement behaviour partnerships like the SUNRISE study [20] can make device-based surveillance much more equitable. SUNRISE depends on the sharing of accelerometry equipment and expertise in accelerometer use. A major barrier to equipment sharing in the SUNRISE study, reflected by respondents in our surveys, has been the difficulties (cost, time, customs clearance, loss of or damage to devices) associated with shipping equipment across international borders and that is a practical problem future regional or global surveillance hubs would face.

Alternative models of movement behaviour surveillance that do not depend on national health surveys might also be very informative. For example, the FitBack initiative https://www.fitbackeurope.eu/en-us/ has provided valuable national and international surveillance of child and adolescent physical fitness, based on measurements made mainly in physical education classes [1, 29]. This model might be considered more widely for countries where school attendance is universal. Other non-traditional approaches to surveillance might also be helpful, e.g. greater use of ‘Citizen Science’ [30]. Citizen Science surveillance initiatives might involve community members using smartphone apps to track and report their own or their child’s physical activity, sedentary behaviour or sleep patterns. These approaches harness the collective power of diverse perspectives and large-scale data collection, potentially offering real-time, contextualized insights into movement behaviours within specific communities, combining device-measured quantitative information and self/proxy-reported qualitative information on the setting, contex and type of behaviour/activity. The validity and reliability of citizen science-derived results are bolstered through meticulous validation protocols, rigorous data quality checks and collaborative efforts between scientific experts and participants, and would require actions to develop standardised methodologies, cross-verification processes and quality assurance [30].

Regardless of the model chosen, surveillance systems should generate good quality information that can drive the development of effective actions in policy and practice [31]. A greater focus on the surveillance of specific domains and contextual factors (e.g. active transportation, active/outdoor play) might be more practical than surveillance of time spent in moderate-to-vigorous-intensity physical activity (MVPA). That type of approach would be informative for population-level monitoring of trends over time and understanding inequalities and responses to changes in policy. A shift in emphasis to surveillance of domains and contexts would also allow countries to focus on the domains that are of highest priority to them. Our respondents emphasised the importance of avoiding a narrow focus, on surveillance of MVPA only for example. They recommended that surveillance is not restricted to individual data, but that actions are required to expand the measurement and monitoring of the ‘upstream’ influences on the movement behaviours (e.g., parent/peer/school and policy environment) that is lacking in many national and international movement behaviour surveillance systems and initiatives at present [1, 3].

Respondents recommended establishing more effective national partnerships to promote child and adolescent movement behaviours in the public health and sports science/sports medicine agendas. This would include increasing policymaker and practitioner awareness and use of WHO guidelines and strategies [4, 5, 10] and more emphasis on policy implementation in relation to the movement behaviours in childhood and adolescence. These improvements will need advocacy for movement behaviours by researchers and an increase in knowledge exchange between experts in surveillance and child and adolescent movement behaviours and policymakers and practitioners in health and other relevant areas (e.g. education, childcare, transport, planning). The AHKGA Global Matrix initiative has shown that physical activity policy development is generally positive, but policy implementation and evaluation are lacking in many countries [11]. The present study shows the high priority placed by experts in surveillance on the need for greater policymaker and practitioner awareness of the importance of child and adolescent movement behaviours, particularly on issues already high on the global public health agenda: NCD prevention, child and adolescent development and the right of children to play [1].The WHO Global Status Report on Physical Activity in 2022 highlighted the need for well-implemented policy on physical activity in all countries, underpinned by robust surveillance [32], anchored to the Sustainable Development Goals.

Substantive changes in surveillance are clearly required globally, but these might be more achievable than one might think with a harmonised approach that builds on international partnerships. For example, existing international surveillance initiatives for the movement behaviours and/or related health outcomes in childhood and adolescence (e.g., SUNRISE, the AHKGA Global Matrix, FitBack) already include many of the elements required for high quality surveillance [1]. These initiatives make use of international collaboration to share expertise and measurement equipment, build human capacity in surveillance and discipline expertise and they support regional and national surveillance via regional or international knowledge exchange/research hubs/peer support networks. Initiatives of this kind could be expanded and made more sustainable by long-term funding and partnership with key existing organisations (e.g., WHO Geneva, regional WHO offices) to sustain surveillance.

The WHO ‘STEPwise approach to surveillance’ (STEPS) [33] does not use methods which would be applicable to movement behaviour surveillance in the age groups covered by the present study, but the approach taken by WHO STEPS to develop a global surveillance system might prove useful as a model for future global surveillance in children and adolescents. STEPS is a globally comparable, standardised and integrated surveillance tool by which countries can collect, analyse and disseminate core information on NCDs across a wide range of environmental and cultural settings. STEPS has succeeded largely via a combination of funding, and support/co-ordination from regional and international WHO offices, providing a good example of the kind of shared international infrastructure and collaboration required for more informative global surveillance of the movement behaviours of children and adolescents [32, 33]. Developing a STEPS-like international system will be much more challenging in some countries than others as engagement with existing WHO-based surveillance of movement behaviours and NCD risk factors varies greatly between countries, but success in some countries provide hope [34] https://www.who.int/teams/noncommunicable-diseases/surveillance/data.

Advances in surveillance will allow regional, national and global stakeholders to confidently identify and respond to trends in movement behaviours and inequalities in these behaviours [32, 35]. Improved surveillance would also allow stakeholders to assess the impact of policy change or societal change, for example, the impacts of climate change, economic crises, political instability and pandemic mitigation measures [11, 36]. Greater collaboration between experts in movement behaviours and policymakers might also prevent unintended harms from policy, or at least identify unintended policy consequences. For example, in Canada, a well-meaning policy, the ‘Child Fitness Tax Credit’, a $500 tax credit for registering a child in an eligible physical activity programme, had limited impact other than increased socio-economic inequalities in health [37], also illustrating the need for more robust evaluation of movement behaviour-related policy. The process of developing higher quality surveillance systems is not ‘just’ about monitoring as a passive data collection exercise. Surveillance tends to reveal previously unnoticed public health problems, which in turn tends to lead to new policy or more informed policy [1]. Researchers could also develop much better-informed research questions if surveillance was of higher quality.

**Fig. 4:**
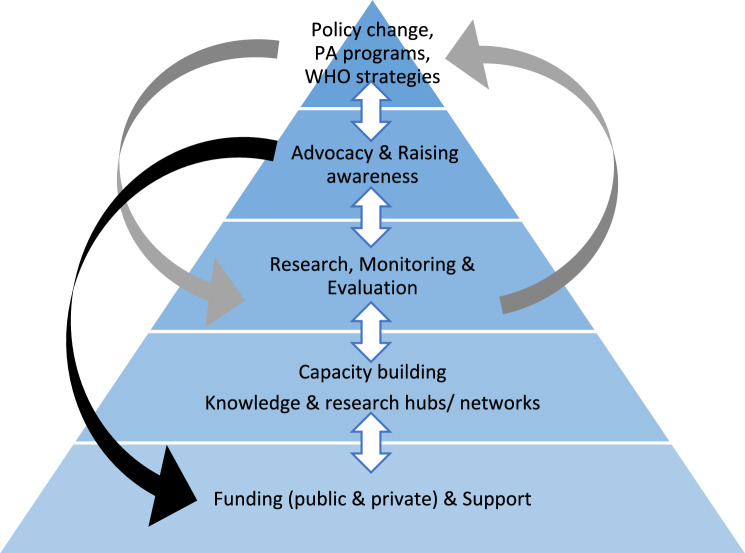
Conceptual model for improving national and international surveillance and achieving a society with more active children and adolescents

Figure 4 illustrates how the present study findings might lead to actions which improve national and international surveillance. The foundation of the conceptual model in Fig. 4 is increased funding for surveillance. The model is not a simple linear pyramid but incorporates feedback loops. For example, more effective researcher advocacy is likely to be needed to achieve increased funding for surveillance, and to ensure that improved surveillance translates to meaningful changes in policy and practice. More advocacy for movement behaviour funding and surveillance may be uncomfortable for movement behaviour researchers, but merits greater effort. A number of the priorities identified in the present study might be addressed by more effective advocacy, e.g. greater policymaker awareness of WHO guidelines and strategies, and of the linkage between the movement behaviours and NCD prevention. There is increasing expectation in HICs that research funding will include knowledge exchange, and that the work of the researcher does not end when the paper is published [38]. Capacity for knowledge exchange in public health is limited in LMICs at present, but there are useful examples [39], and guidance on developing more effective knowledge exchange is available [40]. Successful movement behaviour surveillance research and knowledge exchange can occur within the same project [37] and greater effort in knowledge exchange can have impact in public health policy and practice, including in physical activity and health [41, 42]. One likely reason for public underfunding of child and adolescent movement behaviour surveillance and policy action, even in HICs with well-established surveillance systems such as Canada, is that these behaviours are being perceived as the responsibility not of government or society, but of the individual/family [43]. Future advocacy and knowledge exchange might therefore emphasise the importance of ‘upstream’ socio-ecological influences, above the level of the individual/family, and the key role for public policy in shaping the socio-ecological environment [44].

#### Funding Issues Other than the Need for Increased Funding

Increased and sustained funding is the foundation of improved national and international movement behaviour surveillance, but funding was also the only survey item with marked disagreement between participants, both in their ratings and comments. While 93% of respondents agreed that greater public funding was either ‘very important’ or ‘extremely important’, only 26% felt that it was either very or extremely important to seek funding from private/commercial sources. A number of strongly held concerns were expressed over accepting funding from commercial organisations with interests counter to public health such as tobacco, sugar, infant formula and soft drinks. Funding from commercial organisations may provide an opportunity to advance the current inadequate state of national and international movement behaviour surveillance, but brings a risk of bias [45, 46]. Private funding can lead some to dismiss the findings of research or surveillance funded by commercial organisations, regardless of the efforts made to minimise bias. ‘Big Food’ has undoubtedly tried to delay or even avoid progressive public health policies [47]. There are less obvious vested interests in movement behaviours (‘Big Tech’ or ‘Big Cars’) [48], which might encourage an excessive focus on food as the main source of our major public health problems.

The reality for many respondents to the present study is that private/commercial funding of surveillance or surveillance research is the only option, and this does not just apply to LMICs. There may be no choice between commercial and non-commercial funding, but a decision about whether to conduct surveillance or surveillance research or not. There is potential for commercial vested interests to take advantage of gaps in funding for movement behaviour surveillance and research. Accepting funding for movement behaviour surveillance research should not be in conflict with other public health objectives (e.g. diet or substance use related). It seems possible to carry out successful movement behaviour surveillance projects funded by private donors which do not lead to public health conflicts [49]. However, sustainable surveillance is likely to require government funding in most countries. Some respondents in the present study commented that striking a balance between academic and commercial interests that do not disadvantage public health is what is desirable and felt that striking such a balance was realistic. Intolerance of movement behaviour research and surveillance that is funded from commercial sources may also enforce a Westernised lens on a global issue. Countries have unequal availability of public and non-governmental organisation funding for research and failing to accept commercial funding if it is the only source of funding might lead to increasing inequality in global movement behaviour research and surveillance. Finally, some respondents noted that public/governmental funding brings its own problems such as pressure to avoid criticism of government policy or lack of government policy, or loss of editorial independence.

Regardless of the source of funding, unbiased population health research is highly beneficial to society. The level of disagreement over funding sources observed in the present study suggests that there may be a need to establish a globally accepted framework for future surveillance and surveillance research. This could include a written agreement that the funder will not influence the design and methods, will not be involved in data interpretation and reporting, will not own the data and will not use the funding of surveillance to promote harmful products. Some WHO guidance on the use of commercial funding is available [50] and useful models for managing funding from private/commercial sources for nutrition research are also emerging [51, 52]. Movement behaviour researchers have a responsibility to keep advocating for increased funding for surveillance, to share their research findings widely with relevant stakeholders, and to demonstrate the economic and other benefits which will derive from movement behaviour surveillance. Future research could examine which commercial sources are most acceptable to surveillance researchers (e.g., food versus banking versus insurance), while also establishing best practice guidelines when engaging in private/commercially sponsored research, similar to recent efforts from nutrition researchers [52].

### Study Strengths and Weaknesses

Our study presents strengths that include novelty, timeliness and importance of the topic–particularly in the wake of the adverse impact of the COVID-19 pandemic on the movement behaviours of children and adolescents [14–19] and with relatively recent evidence-based 24-h guidelines for the movement behaviours from birth to the end of adolescence [5–10]. High-quality, evidence-based movement behaviour guidelines are not matched by high quality global surveillance at present [3]. A further strength is the wide global reach in terms of geographical representation and level of economic development, almost equal numbers of respondents from LMICs/HICs and equal numbers of women and men, which is very rare in this field of research [53]. Respondents drew on expertise across the age range from early childhood to the end of adolescence and response rates from the two international initiatives were fairly high. Lastly, our findings suggest that there are many important research and surveillance gaps, including the need for a framework with which to use private/commercial funding and the need for better surveillance measures of screen time and physically active play.

Weaknesses of the present study include the restriction of inclusion to SUNRISE and AHKGA Report Card participants. This decision was taken largely on practical grounds given that these two global initiatives provided an expert constituency that was easily identifiable and relatively easy to contact and would prevent the over-representation from any participating countries or regions. Since both initiatives are global, the inclusion of respondents from SUNRISE and AHKGA ensured wide geographical reach and avoided the under-representation of LMICs, typical of most biomedical research. SUNRISE and AHKGA focus on 3–17-year-olds, so different priorities could have been identified for toddlers and infants. However, many of the improvements suggested by respondents in the present study (e.g. improved human capacity, better understanding of the movement behaviours and how to measure them, increased funding of surveillance, more sophisticated methods) would likely apply to surveillance in infants and toddlers too [1, 3]. In addition, many of the survey respondents also have expertise in movement behaviour surveillance in infants and toddlers. Restricting inclusion to those involved in the SUNRISE and AHKGA initiatives may have limited generalisability though; for example, responses may have differed from researchers who work outside the initiatives, including those who work exclusively in physical activity (not the other movement behaviours), or in adults rather than children and adolescents.

While the sample size for the present study was large relative to the global pool of paediatric movement behaviour surveillance experts, and for Delphi studies based on international expertise, the sample size for statistical comparisons was small. This may have limited our ability to detect differences, but Delphi Studies aim to identify consensus and statistical inferences are usually of secondary importance [20–22, 54]. We used a modified Delphi Study with items offered to participants that were identified by the international leadership group of the study prior to the round 1 survey rather than being completely open at the start of the process. The modified Delphi design is now relatively common [25] and was intended to produce a more efficient process than a traditional Delphi design in the present study, but it may have constrained survey responses. However, participants were invited to suggest additional items to those provided to them at round 1, and only one new item was suggested and added (the linkage of movement behaviours to academic attainment, categorised under other issues). There was also possible acquiescence bias in responses as the study leadership group included those who lead the SUNRISE and AHKGA initiatives, though participants were informed that individual survey responses would be treated as anonymous and not be shared with the present study leadership group in an effort to minimise this source of bias.

## Conclusions

This study fulfils an important practical purpose in identifying the actions required for the desired transformation in the surveillance of movement behaviours throughout childhood and adolescence. The findings underscore the importance of several specific actions, namely increased funding, enhanced human capacity, improved technology accessibility and methods, and greater use of support networks. By implementing these actions, the transformative changes envisioned for movement behaviour surveillance [1, 3] can become more attainable. The present study also suggests that experts wish to avoid a narrow surveillance focus, for example only on MVPA, and that guidance on the use of commercial and government funding in physical activity research and surveillance is required.

## Supplementary Information

Below is the link to the electronic supplementary material.

Supplementary file1 (DOCX 46 KB)

## Declarations

### Availability of Data

The anonymised data from survey rounds 1 and 2 are available from the corresponding author upon reasonable request.
