# Correction to: Improving National and International Surveillance of Movement Behaviours in Childhood and Adolescence: An International Modified Delphi Study

**DOI:** 10.1007/s40279-025-02185-7

**Published:** 2025-04-02

**Authors:** John J. Reilly, Rachel Andrew, Chalchisa Abdeta, Liane B. Azevedo, Nicolas Aguilar Farias, Sharon Barak, Farid Bardid, Bruno Bizzozero-Peroni, Javier Brazo-Sayavera, Jonathan Y. Cagas, Mohamed-Souhaiel Chelly, Lars B. Christiansen, Visnja D. Djordjic, Catherine E. Draper, Asmaa El-Hamdouchi, Elie-Jacques Fares, Aleš Gába, Kylie D. Hesketh, Mohammad Sorowar Hossain, Wendy Huang, Alejandra Jáuregui, Sanjay K. Juvekar, Nicholas Kuzik, Richard Larouche, Eun-Young Lee, Sharon Levi, Yang Liu, Marie Löf, Tom Loney, José Francisco López-Gil, Evelin Mäestu, Taru Manyanga, Clarice Martins, Maria Mendoza-Muñoz, Shawnda A. Morrison, Nyaradzai Munambah, Tawonga W. Mwase-Vuma, Rowena Naidoo, Reginald Ocansey, Anthony D. Okely, Aoko Oluwayomi, Susan Paudel, Bee Koon Poh, Evelyn H. Ribeiro, Diego Augusto Santos Silva, Mohd Razif Shahril, Melody Smith, Amanda E. Staiano, Martyn Standage, Narayan Subedi, Chiaki Tanaka, Hong K. Tang, David Thivel, Mark S. Tremblay, Edin Uzicanin, Dimitris Vlachopoulos, E. Kipling Webster, Dyah Anantalia Widyastari, Pawel Zembura, Salome Aubert

**Affiliations:** 1 Department of Psychological Sciences and Health, University of Strathclyde, Glasgow, Scotland; 2 School of Education, University of Wollongong, Wollongong, Australia; 3 School of Sport and Physical Activity, Sheffield Hallam University, Sheffield, UK; 4 Department of Physical Education, Sports and Recreation, Universidad de La Frontera, Temuco, Chile; 5 Department of Nursing, Faculty of Health Science, Ariel University, Ariel, Israel; 6 Institute of Education, University of Strathclyde, Glasgow, Scotland; 7 Instituto Superior de Educación Física, Universidad de La República, Rivera, Uruguay; 8 Health and Social Research Center, Universidad de Castilla-La Mancha, Cuenca, Spain; 9 Department of Sports and Computer Science, Universidad Pablo de Olavide, Seville, Spain; 10 PDU EFISAL, Centro Universitario Regional Noreste, Universidad de La República, Montevideo, Uruguay; 11 Department of Sports Science, College of Human Kinetics, University of the Philippines Diliman, Quezon, Philippines; 12 Research Laboratory (LR23JS01) «Sport Performance, Health and Society», Higher Institute of Sport and Physical Education of Ksar Saîd, University of Manouba, Tunis, Tunisia; 13 Department of Sport Sciences and Clinical Biomechanics, University of Southern Denmark, Odense, Denmark; 14 Faculty of Sport and Physical Education, University of Novi Sad, Novi Sad, Serbia; 15 Faculty of Health Sciences, SAMRC Developmental Pathways for Health Research Unit, University of the Witwatersrand, Johannesburg, South Africa; 16 National Centre for Energy Sciences and Nuclear Techniques, Kenitra, Morocco; 17 American University of Beirut, Beirut, Lebanon; 18 Faculty of Physical Culture, Palacký University Olomouc, Olomouc, Czech Republic; 19 Institute of Physical Activity and Nutrition, Deakin University, Burwood, Australia; 20 Biomedical Research Foundation, Dhaka, Bangladesh; 21 Independent University, Dhaka, Bangladesh; 22 Department of Sport, Physical Education and Health, Hong Kong Baptist University, Hong Kong, China; 23 Department of Physical Activity and Healthy Lifestyles, National Institute of Public Health, Cuernavaca, México; 24 Vadu Rural Health Program, KEM Hospital Research Centre, Pune, India; 25 Children’s Hospital of Eastern Ontario Research Institute, Ottawa, ON K1H 5B2 Canada; 26 Faculty of Health Sciences, University of Lethbridge, Lethbridge, Canada; 27 School of Kinesiology and Health Studies, Queen’s University, Kingston, Canada; 28 Efsharibari- National Program for Active and Healthy Living, Ministry of Health, Haifa, Israel; 29 School of Public Health, University of Haifa, Haifa, Israel; 30 School of Physical Education, Shanghai University of Sport, Shanghai, China; 31 Department of Biosciences and Nutrition, Karolinska Institutet, Solna, Sweden; 32 College of Medicine, Mohammed Bin Rashid University of Medicine and Health Sciences, Dubai Health, Dubai, United Arab Emirates; 33 One Health Research Group, Universidad de Las Américas, Quito, Ecuador; 34 Institute of Sport Sciences and Physiotherapy, Faculty of Medicine, University of Tartu, Tartu, Estonia; 35 Division of Medical Sciences, University of Northern British Columbia, Prince George, Canada; 36 Faculty of Sports, Research Centre of Physical Activity, Health and Leisure, Porto, Portugal; 37 Laboratory for Integrative and Translational Research in Population Health (ITR), University of Porto, Porto, Portugal; 38 Faculty of Sport Sciences, University of Extremadura, Badajoz, Spain; 39 Human Potential Translational Research Programme, Yong Loo Lin School of Medicine, National University of Singapore, Singapore, Singapore; 40 Faculty of Medicine and Health Sciences, University of Zimbabwe, Harare, Zimbabwe; 41 Centre for Social Research, University of Malawi, Zomba, Malawi; 42 Discipline of Biokinetics, Exercise and Leisure Sciences, College of Health Sciences, University of KwaZulu-Natal, Durban, South Africa; 43 School of Education and Leadership Studies, University of Ghana, Legon, Ghana; 44 School of Health and Society, University of Wollongong, Wollongong, Australia; 45 Human Kinetics and Health Education, University of Lagos, Lagos, Nigeria; 46 Institute for Physical Activity and Nutrition, School of Exercise and Nutrition Sciences, Deakin University, Burwood, Australia; 47 Centre for Community Health Studies (ReaCH), Faculty of Health Sciences, University Kebangsaan Malaysia, Kuala Lumpur, Malaysia; 48 Evelyn H. Ribeiro, Physical Activity Epidemiology Group at the University of Sao Paulo (GEPAF/USP), Sao Paulo, Brazil; 49 Department of Physical Education, Sports Center, Federal University of Santa Catarina, Santa Catarina, Brazil; 50 Centre for Healthy Ageing and Wellness (HCARE), Universiti Kebangsaan Malaysia, Bandar Baru Bangi, Malaysia; 51 School of Nursing, The University of Auckland, Auckland, New Zealand; 52 Pennington Biomedical Research Center, Louisiana State University, Louisiana, USA; 53 Centre for Motivation and Health Behaviour Change, Department for Health, University of Bath, Bath, UK; 54 The Lown Scholar, Harvard T.H. Chan School of Public Health, Harvard University, Cambridge, USA; 55 Department of Human Nutrition, Tokyo Kasei Gakuin University, Tokyo, Japan; 56 Faculty of Public Health, Pham Ngoc Thach University of Medicine, Ho Chi Minh City, Vietnam; 57 Laboratory of the Metabolic Adaptations to Exercise under Physiological and Pathological Condition (AME2P UPR3533), International Research Chair “Health in Motion”, Clermont Auvergne University, Clermont-Ferrand, France; 58 Healthy Active Living and Obesity Research Group, Children’s Hospital of Eastern Ontario Research Institute, Ottawa, ON Canada; 59 Faculty of Physical Education and Sport, University of Tuzla, Tuzla, Bosnia-Herzegovina; 60 Department of Sport and Health Science, University of Exeter, Devon, UK; 61 Department of Kinesiology, Recreation, and Sport Studies, University of Tennessee, Knoxville, USA; 62 Institute for Population and Social Research, Mahidol University, Nakhon Pathom, Thailand; 63 Jozef Pilsudski University of Physical Education, Warsaw, Poland; 64 Active Healthy Kids Global Alliance, Healthy Active Living and Obesity Research, Ottawa, Canada; 65 Department of Occulational and Physical Therapy, University of Namibia, Windhoek, Namibia

**Correction to** **:** **Sports Medicine (2025) 55:203–219** 10.1007/s40279-024-02104-2

In this article the Co-author’s name José Francisco López-Gil was incorrectly written as Jose Francisco Lopez Gil.

José Francisco López-Gil’s affiliation One Health Research Group, Universidad de Las Américas, Quito, Ecuador was incorrectly written as Centro de Investigacion Biomedica, Unidad de Actividad Fisica Infanto-Juvenil, University of Navarra, Pamplona, Spain.

The original article has been updated accordingly.

