# Supplementary material for: Improving National and International Surveillance of Movement Behaviours in Childhood and Adolescence: An International Modified Delphi Study
Source: Sports Med. 2024 Oct 3;55(1):203–19. doi: 10.1007/s40279-024-02104-2 (PMC11787202; doi:10.1007/s40279-024-02104-2)
Supplement: Supplementary file 1 — Supplementary file1 (DOCX 46 KB) [file 40279_2024_2104_MOESM1_ESM.docx]

**Supplementary Files**

**File 1 1.** Round 2 Questionnaire.

Note. Questions 6-40 all displayed the options shown in question 5.

**SOME BASIC INFORMATION ABOUT YOU**

Q1. What country do you represent?

________________________________________________________________

Q2. What sector do you work in most?

- Academia- graduate or doctoral or postdoctoral researcher (1)
- Academia- academic post (2)
- Government/Policy (3)
- Non-governmental organization (4)
- Other (5)

Q3. Can you please tell us your gender

- Male (1)
- Female (2)
- Other (3)
- Prefer not to say (4)

Q4. Do you represent Sunrise or AHKGA or both Sunrise and AHKGA?

- Sunrise (1)
- AHKGA (2)
- Both Sunrise and AHKGA (3)

Could you please provide your email address

________________________________________________________________

**FUNDING** FOR HIGH QUALITY NATIONAL SURVEILLANCE AND GLOBAL SURVEILLANCE

 FOR EACH QUESTION BELOW CAN YOU PLEASE RESPOND BY VOTING YOUR RATING OF IMPORTANCE

Q5. More funding for improved surveillance at national level

- Don't know/prefer not to say (1)
- Not at all important (2)
- Slightly important (3)
- Moderately important (4)
- Very important (5)
- Extremely important (6)

Q6. More funding for improved surveillance at trans-national level (e.g., regional or global surveillance)

Q7. More funding for research on surveillance (e.g., methodology)

Q8. Funding from public (government) sources

Q9. Funding from charitable/non-governmental sources

Q10. Funding from private sources (e.g., corporations, commercial sources)

Q11. Is funding from private sources which harm public health an important concern for you (e.g., funding from tobacco, sugar, soft drinks companies)?

Q12. Do you want to expand on your responses above, or comment on other funding issues not listed above which you feel are important?

________________________________________________________________

**TRAINING AND CAPACITY BUILDING** FOR HIGH QUALITY NATIONAL AND GLOBAL SURVEILLANCE

 FOR EACH QUESTION BELOW CAN YOU PLEASE RESPOND BY VOTING YOUR RATING OF IMPORTANCE

Q13.Capacity building-human capacity- in physical activity and health (e.g. improved education, training, and awareness among health professionals and policymakers)

Q14. Other forms of capacity building (e.g., improved infrastructure/facilities/equipment for surveillance)

Q15. Centralised global or regional hubs which would support data collection, quality control, of data, data visualisation (e.g., through online programmes, through partnerships such as AHKGA or SUNRISE; like WHO STEPS for adults)

Q16. Do you want to expand on your responses above, or comment on other training/capacity building issues not listed above which you feel are important?

________________________________________________________________

**METHODOLOGY** FOR HIGH QUALITY NATIONAL AND GLOBAL SURVEILLANCE IN THE UNDER 5s, CHILDREN, AND ADOLESCENTS

 FOR EACH QUESTION BELOW CAN YOU PLEASE RESPOND BY VOTING YOUR RATING OF IMPORTANCE

Q17. Use of globally valid and reliable standardized, but culturally-adapted, measurement protocols for measurement of behaviours (e.g. like the SUNRISE protocol for 3-4 year olds)

Q18. Surveys based on standardised sampling **method**, with representative (or broadly representative) samples (e.g. like WHO STEPS in adults)

Q19. Surveys large enough to provide confident estimates of prevalence estimates and trends, large enough to identify inequalities, and with stratification by socio-economic status and urban-rural settings

Q20. Greater inclusion of groups currently under-represented in national and global surveillance (e.g., the Under 5s; with chronic diseases and/or disability; from ethnic, indigenous, or religious minorities; rural as well as urban areas; not attending school)

Q21.Improved methodology for measurement of **total time** spent in physical activity (e.g. light intensity, MVPA)

Q22. Improved methodology for measurement of time spent in sedentary behaviour, including recreational screen time

Q23.The ability to measure **time spent in the following physical activity domains** :

(A) Active play

(B) Active transportation

(C) Organised sport and physical activity

(D) Physical education

(E) Chores

(F) Work/occupation/labour

Other (please state)

________________________________________________________________

Q24. The ability to measure time spent in all or many child and adolescent sedentary behaviour domains:

(A) Time spent restrained (e.g., in highchairs, strollers, car seats)

(B) TV time

(C) Time spent in front of other screens (e.g., mobiles, laptops, tablets)

(D) Time spent using screens for games and recreation

(E) Screen time before bedtime

(F) Screens for academic time e.g., schoolwork/homework

Other (please state)

Q25. The ability to measure the **‘dose’ (e.g., frequency x intensity x duration) for each physical activity domain** you selected in the previous question Q21

Q26. The ability to measure **the context for each physical activity domain** selected in Q 21 (e.g., level of enjoyment, type of sport or organized activity, indoors vs outdoors, alone or with others)

Q27. The ability to measure the **context for each sedentary behaviour domain** selected in Q22 above (e.g., level of enjoyment, alone or with others)

Q28. Improved methods for measuring **influences** on physical activity, sedentary behaviours, and sleep:

(A) Environment and community **influence**

(B) Family and peers **influence**

(C) School/pre-school/childcare centres **influence**

(D) **Influence** of Habits and cultural norms

(E) **Influence** of Government and policies

Other (please state)

Q29. Improved methods for measuring **outcomes** of the behaviours (e.g., healthy body weight or healthy body fatness, cognitive and academic outcomes, physical fitness, mental health)

Q30. Improved methods of measurement of ‘vigorous-intensity aerobic activities, as well as those that strengthen muscle and bone'

Q31. Use of widely available consumer technology for surveillance (e.g., smartphones, wearables)

Q32. Do you want to expand on your responses above, or comment on other methodological issues not listed above which you feel are important?

________________________________________________________________

**OTHER ISSUES** REQUIRED TO ACHIEVE HIGH QUALITY NATIONAL AND GLOBAL SURVEILLANCE

 FOR EACH QUESTION BELOW CAN YOU PLEASE RESPOND BY NOTING YOUR RATING OF IMPORTANCE

Q33. Endorsement by, or support of, WHO, UNICEF, International Olympic Committee or other global organisations

Q34. Greater policymaker awareness of relevant WHO guidelines, programmes and strategies (e.g., Guidelines for Under 5 years; Guidelines for School-Aged Children and Adolescents; Global Action Plan on Physical Activity).

Q35. National guidelines (e.g., based on WHO global guidelines) for time spent in physical activity guidelines or 24-hour movement behaviour guidelines

Q36. Availability of policies/ programmes/ recommendations which policymakers can adopt to promote physical activity/related behaviours (or greater awareness of existing policies and programmes)

Q37. Better partnerships between members of surveillance research/advocacy organisations (like Sunrise and AHKGA) and policymakers at nationally or international level

Q38. Greater emphasis on ensuring equity between the global north-south in how global surveillance is governed, carried out, analysed and published

Q39. Better articulation and recognition of the **co-benefits** of physical activity, and links to other issues which are high on the global agenda:

(A) COVID-19 recovery

(B) The Sustainable Development Goals

(C) Non-Communicable Disease Prevention

(D) Climate Change

(E) The Right of the Child to Play

(F) Child & Adolescent Development (e.g., social, emotional, cognitive development)

(G) Malnutrition in all its forms (e.g., underweight, overweight and obesity)

**(H) ACADEMIC ATTAINMENT**

Q40. Any other issues not listed above you think are essential?

________________________________________________________________

**Supplementary File** **2.** **Top Two Priorities (rating median, range) by Category of Response between Low-and Middle-Income Country (LMIC) and High Income Country (HIC) Respondents – Round 2**

| Category | Item | LMIC  (n=29) | | HIC  (n=30)) | | P-value* |
| --- | --- | --- | --- | --- | --- | --- |
|  |  | Median | Range | Median | Range |  |
| Funding | More national funding | 5 | 3-5 | 5 | 3-5 | 0.15 |
|  | Public/government funding | 5 | 3-5 | 5 | 3-5 | 0.93 |
| Capacity Building | Improved human capacity | 5 | 3-5 | 5 | 3-5 | 0.90 |
|  | Central or regional training hubs | 4 | 3-5 | 4 | 3-5 | 0.52 |
| Methods | Standardised protocols | 5 | 4-5 | 5 | 4-5 | 0.42 |
|  | Ability to measure time spent with screens other than TV | 5 | 4-5 | 5 | 4-5 | 0.80 |
| Other Issues | Greater national use of existing policies/programmes /strategies | 5 | 4-5 | 5 | 4-5 | 0.72 |
|  | Link to NCD Prevention | 5 | 4-5 | 5 | 4-5 | 0.57 |

*Differences between medians from LMICs and HICs tested for significance by Mann-Whitney U Tests. Rating from 1.0-5.0, 5 highest,1 lowest.

**Supplementary File 3.Top Two Priorities (rating median, range) by Category of Item, Between SUNRISE Study, Active Healthy Kids Global Alliance (AHKGA) and Both SUNRISE and AHKGA Respondents – Round 2**

| Categories | Items | SUNRISE  (n=20) | | AHKGA  (n=26) | | | Both  (n=13) | | | P*-value |
| --- | --- | --- | --- | --- | --- | --- | --- | --- | --- | --- |
|  |  | Median | Range | Median | | Range | Median | Range | |  |
| Funding | More national funding | 5 | 4-5 | 5 | | 4-5 | 5 | | 4-5 | 0.85 |
|  | Public/government funding | 5 | 4-5 | 5 | 4-5 | | 5 | | 4-5 | 0.30 |
| Capacity Building | Improved human capacity | 5 | 4-5 | 5 | 4-5 | | 5 | | 4-5 | 0.45 |
|  | Central or regional training hubs | 5 | 4-5 | 4 | 4-5 | | 4 | | 4-5 | 0.66 |
| Methods | Standardised protocols | 5 | 4-5 | 5 | 4-5 | | 5 | | 4-5 | 0.59 |
|  | Ability to measure time spent with other screens | 4 | 3-5 | 5 | 4-5 | | 5 | | 4-5 | **0.07** |
| Other Issues | Greater national use of existing policies/programmes /strategies | 4 | 3-5 | 5 | 4-5 | | 5 | | 4-5 | 0.37 |
|  | Link to NCD Prevention | 5 | 3-5 | 5 | 4-5 | | 5 | | 4-5 | 0.39 |

*Differences between medians tested for significance by Kruskal Wallis Tests. Respondent ratings ranged from 1.0-5.0, 5 highest.

**Supplementary File 4.** Summary results from round 1 shown to participants prior to second round completion.

**FUNDING SECTION**

| Item | % Very-Extremely Important | Mean Score | SD |
| --- | --- | --- | --- |
| More national funding | 95.0 | 4.5 | 0.7 |
| More transnational funding | 79.7 | 4.1 | 1.0 |
| More surveillance research funding | 84.8 | 4.1 | 0.9 |
| Government funding | 93.1 | 4.4 | 0.8 |
| Charitable/Non Governmental | 50.0 | 3.6 | 1.0 |
| Private/commercial funding | 32.8 | 2.9 | 1.2 |
| Concerns re commercial funding from sources harmful to health | 79.7 | 4.0 | 1.3 |

**CAPACITY BUILDING SECTION**

| Item | % Very-Extremely Important | Mean Score | SD |
| --- | --- | --- | --- |
| Improved human capacity | 91.7 | 4.6 | 0.7 |
| Other capacity-infrastructure, equipment | 78.3 | 4.1 | 0.8 |
| Central or regional hubs to improve/support capacity | 86.7 | 4.4 | 0.7 |

**METHODOLOGY SECTION**

| Item | % Very-Extremely important | Mean | SD |
| --- | --- | --- | --- |
| Standard protocols like Sunrise | 96.6 | 4.7 | 0.5 |
| Standardised sampling methods | 84.5 | 4.2 | 0.7 |
| Surveys large enough to identify inequalities | 94.8 | 4.5 | 0.6 |
| Greater inclusion of groups currently under-represented | 86.2 | 4.3 | 1.1 |
| Improved methods for time spent in PA (total and/or MVPA) | 86.2 | 4.3 | 0.8 |
| Improved methods for time spent sedentary | 93.1 | 4.5 | 0.7 |
| Ability to measure time spent in active play | 93.1 | 4.5 | 0.7 |
| Ability to measure time spent in active transportation | 82.8 | 4.2 | 0.9 |
| Ability to measure time spent in organised sport and PA | 86.2 | 4.2 | 0.7 |
| Ability to measure time spent in Phys Ed | 86.2 | 4.3 | 0.7 |
| Ability to measure time spent in chores | 55.2 | 3.6 | 1.9 |
| Ability to measure time spent in work | 62.1 | 3.5 | 1.0 |

| Ability to measure time spent restrained | 67.2 | 3.7 | 1.0 |
| --- | --- | --- | --- |
| Ability to measure TV time | 87.9 | 4.4 | 0.8 |
| Ability to measure time spent with other screens | 96.5 | 4.7 | 0.6 |
| Ability to measure recreational screen time | 93.1 | 4.5 | 0.7 |
| Ability to measure screen use before bedtime | 77.2 | 4.2 | 0.9 |
| Ability to measure academic screen time | 81.0 | 4.1 | 0.9 |
| Ability to measure dose of PA domains above | 92.9 | 4.4 | 0.6 |
| Ability to measure context of PA domains above | 79.3 | 4.1 | 0.9 |
| Ability to measure context of SB domains above | 77.6 | 4.1 | 1.0 |

| Improved measurement of the influence of environment/community | 87.9 | 4.3 | 0.7 |
| --- | --- | --- | --- |
| Improved measurement of the influence of family/peers | 87.9 | 4.3 | 0.7 |
| Improved measurement of the  influence of preschool/school | 84.5 | 4.2 | 0.8 |
| Improved measurement of the influence of habits and cultural norms | 87.9 | 4.2 | 0.7 |
| Improved measurement of the influence of government and policy | 93.0 | 4.4 | 0.6 |
| Improved methods for measurement of outcomes | 72.4 | 4.0 | 0.9 |
| Improved measurement of Vigorous intensity PA and muscle/bone strengthening PA | 75.4 | 4.0 | 0.9 |
| Improved methods based on use of consumer technology/wearables | 63.2 | 3.7 | 1.3 |

**OTHER ISSUES RELATED TO IMPROVED SURVEILLANCE SECTION**

| Item | % Very-Extremely Important | Mean Score | SD |
| --- | --- | --- | --- |
| Endorsement of international bodies e.g. WHO | 82.8 | 4.3 | 0.9 |
| Greater policymaker awareness of guidelines/policies/strategies | 94.8 | 4.6 | 0.6 |
| National guidelines | 91.4 | 4.5 | 0.7 |
| Greater national use of existing policies/programmes /strategies | 89.7 | 4.5 | 0.7 |
| Better partnerships | 94.8 | 4.6 | 0.7 |
| Equity north-south | 87.9 | 4.4 | 0.9 |

**LINKAGE OF SURVEILLANCE TO OTHER ISSUES/CO-BENEFITS OF PHYSICAL ACTIVITY SECTION**

| Item | % agreement very or extremely important | Mean | SD |
| --- | --- | --- | --- |
| Link to COVID-19 recovery | 64.9 | 3.9 | 1.0 |
| Link to the SDGs | 81.0 | 4.3 | 0.9 |
| Link to NCD prevention | 85.9 | 4.4 | 1.0 |
| Link to Climate Change | 62.1 | 4.0 | 1.1 |
| Link to Right of Child to Play | 87.9 | 4.3 | 0.7 |
| Link to child/adolescent development | 96.6 | 4.5 | 0.6 |
| Link to malnutrition in all its forms | 84.5 | 4.1 | 0.8 |

**TOP TEN RATED ITEMS ACROSS ALL SECTIONS FROM ROUND 1, RANKED 1-10**

| Item | % agreement very or extremely important | Mean score |
| --- | --- | --- |
| Standard protocols like Sunrise | 96.6 | 4.7 |
| Link to child/adolescent development | 96.6 | 4.5 |
| Ability to measure time spent with other screens | 96.5 | 4.7 |
| More national funding | 95.0 | 4.5 |
| Better partnerships | 94.8 | 4.6 |
| Greater policymaker awareness of guidelines/policies/strategies | 94.8 | 4.6 |
| Surveys large enough to identify inequalities | 94.8 | 4.5 |
| Improved methods for time spent sedentary | 93.1 | 4.5 |
| Ability to measure time spent in active play | 93.1 | 4.5 |
| Government funding | 93.1 | 4.5 |

**BOTTOM FIVE RATED ITEMS ACROSS ALL SECTIONS FROM ROUND 1, RANKED 1-5**

| Item | % agreement very or extremely important | Mean score |
| --- | --- | --- |
| Private/commercial funding | 32.8 | 3.0 |
| Charitable/Non Governmental | 50.0 | 3.6 |
| Ability to measure time spent in chores | 55.2 | 3.6 |
| Ability to measure time spent in work | 62.1 | 3.5 |
| Link to Climate Change | 62.1 | 4.0 |

**END OF ROUND 1 SUMMARY SHARED WITH STUDY PARTICIPANTS PRIOR TO ROUND 2**

**Supplementary File 5**

**TOP TEN RATED ITEMS ACROSS ALL SECTIONS FROM ROUND 2**

| Item | % agreement very or extremely important | Median score out of 5 |
| --- | --- | --- |
| More national funding | 100 | 5 |
| Standard protocols like Sunrise | 100 | 5 |
| Ability to measure time spent with other screens | 98 | 5 |
| Link to NCD prevention | 98 | 5 |
| Government funding | 96 | 5 |
| Greater national use of existing policies/programmes /strategies | 96 | 5 |
| Surveys large enough to identify inequalities | 96 | 5 |
| Greater inclusion of groups currently under-represented | 95 | 5 |
| Better partnerships | 95 | 5 |
| Link to child/adolescent development | 95 | 5 |

**BOTTOM FIVE RATED ITEMS ACROSS ALL SECTIONS FROM ROUND 2**

| Item | % agreement very or extremely important | Median score (range) |
| --- | --- | --- |
| Private/commercial funding | 26 | 4 (1-6) |
| Ability to measure time spent in chores | 39 | 4 (1-6) |
| Ability to measure time spent in work | 42 | 4 (1-6) |
| Link to Climate Change | 44 | 4 (1-6) |
| Link to COVID-19 recovery | 49 | 4 (1-6) |
| Charitable/Non Governmental | 49 | 4 (1-6) |
